# The Chemical Profiling and Anticancer Potential of Functional Polysaccharides from Flos Sophorae Immaturus

**DOI:** 10.3390/molecules27185978

**Published:** 2022-09-14

**Authors:** Wenting Zhong, Chunmiao Yang, Yongze Zhang, Yumeng Liu, Dongsheng Yang

**Affiliations:** 1 College of Pharmacy and Food Science, Zhuhai College of Science and Technology, Zhuhai 519041, China; 2 College of Life Science, Jilin University, Changchun 130012, China

**Keywords:** Flos Sophorae Immaturus, polysaccharide extraction, structural characterization, HCC, SMMC 7721 cells, inhibition of proliferation

## Abstract

Polysaccharides from Flos Sophorae Immaturus (FSI) are one of its pharmacological compounds that can perform effective activities. Aiming to extract the most effective polysaccharides against hepatocellular carcinoma (HCC), the polysaccharides were separated from FSI through ultrasonic microwave extraction, and the first comparison was carried out on the characterization of the structure and its cytotoxic properties on HCC SMMC 7721 cells of undeproteinized purified polysaccharides (PFSI-1) and papain-deproteinized polysaccharides (PFSI-2) from FSI. The findings indicated that PFSI-1 and PFSI-2 had characteristic absorption peaks of polysaccharides; PFSI-1 contained three monosaccharides and PFSI-2 contained ten; and SEM, AFM, and NMR were consistent with the verification of IR polysaccharide characteristics, suggesting probable additional latent activities. The pharmacotoxic effects of both PFSI-1 and PFSI-2 on SMMC 7721 cells (*p* < 0.05), attenuated the migration ability of SMMC 7721 cells (*p* < 0.05) and promoted apoptosis (*p* < 0.05), with an increase in G0/G1-phase cells and decrease in S-phase cells in the PFSI-1 as well as a decrease in G0/G1-phase cells, increase in S-phase cells, and decrease in apoptosis in the PFSI-2 (*p* < 0.05). The significant cytotoxic effect of PFSI-2 on SMMC 7721 cells (*p* < 0.05) and its protective effect on human hepatic L02 cells (HL-7702) at low concentrations (*p* > 0.05) could indicate its potential as a new drug for the treatment of HCC.

## 1. Introduction

Flos Sophorae Immaturus (FSI) refers to a dried flower bud, one of the homologous Chinese medicinal herbs derived from Sophora japonica L. It has been proven that the different activities of FSI reveal that its extracts have specific pharmacological agents, such as hypoglycemic [1], hypotensive [2], antioxidant [3], antiaging [4], antibacterial, antimicrobial [5], antitumor [6], etc. As a traditional Chinese medicine, FSI contains various functional chemical components, including rutin, quercetin, polysaccharides, kaempferol, etc. [7]. The dominant components are flavonoids and polysaccharides, and the flavonoids have been more intensively studied, whereas, due to the molecular weight limitation, the amount of research on polysaccharides from FSI (PFSI) has been rather modest. At the moment, the investigation of the biological activity of polysaccharides has been the focus of research by specialists and scholars at home and abroad with the discovery that plant polysaccharides exhibit certain anti-hepatocellular carcinoma (HCC) activities [8], such as the cultivated Ginseng polysaccharides [9], Ganoderma Lucidum polysaccharides [10], Schisandra Chinensis polysaccharides [11], Astragalus polysaccharides [12], etc. Modern pharmacological studies on the mechanisms of the antitumor role of phyto-polysaccharides [13] have found that phyto-polysaccharides mainly act on hepatocellular tumors by suppressing tumor growth (10), inducing apoptosis [14], enhancing immune functionality [15], and synergizing with chemotherapeutic substances [16]. Despite the advances in clinical practice, the prognosis of HCC remains unsatisfactory in all cases. During the treatment process, almost all patients will be resistant to the treatment. Therefore, seeking new adjuvant drugs to treat HCC can be an attractive way for patients to prolong the length of their lives and raise their quality of life. Globally, HCC stands out as the most common fatal malignancy as well as one of the common onset tumors in China. For more than 90% of all HCCs, chemotherapy, and immunotherapy would be the best therapeutic approaches [17]. In the course of treatment, resistance to drugs occurs in almost all patients. While taking antitumor chemotherapy drugs to kill tumor cells, normal cells are injured somewhat; therefore, locating high-efficiency and low-toxicity antitumor drugs assumes considerable importance, and natural compounds in nature have become one of the available vehicles for patients to prolong life and increase the quality of life.

Phyto-polysaccharides have some anti-HCC activity and both undeproteinated and deproteinated polysaccharides have anti-HCC activity [18]. According to reports [19], polysaccharides exert their antitumor properties through antioxidant activity, immune-enhancing activity, reinforcing the efficacy of chemotherapeutic agents, and alleviating side effects. PFSI has some antioxidant activity and can be used as a natural antioxidant. The use of antioxidants as functional food ingredients and dietary supplements, both natural and synthetic, has gained popularity in recent years. Because there is an unbalanced ratio of antioxidants to free radicals, oxidative reactions can harm proteins, lipids, and DNA. Interest in antioxidants has increased as a result of their ability to prevent oxidative deterioration in food and pharmaceutical products as well as in the body and against disease processes brought on by oxidative stress. For screening the antioxidant capacities of plants and compounds produced from plants, appropriate methods that focus on the kinetics of the reactions involving the antioxidants and address the mechanism of antioxidant activity are required. Numerous investigations into the relationship between food and human health, using various samples of research interest, have been conducted [20,21]. In addition, FSI extensions have antitumor properties that are concentration- and dose-dependent and can be substantially boosted by combining them with anti-HCC drugs [22]. In all these cases, PFSI might be a potentially efficacious and nontoxic alternative antitumor or adjuvant drug for the treatment of tumors by compounds found in nature.

Within the context of the proposed study, the monosaccharide composition and structural characterization of PFSI were firstly investigated by isolating and purifying the polysaccharides, which could lead to a quantitative bioactivity enhancement of the polysaccharides [23]. Samples were isolated and purified by the undeproteinated (PFSI-1) and 80 U/mL papain deproteinization methods (PFSI-2). The characteristics of the above polysaccharides were analyzed by utilizing IR, SEM, AFM, and NMR. To conclude, the inhibitory role of PFSI-1 and PFSI-2 on the proliferation of HCC cells (SMMC 7721) was compared, while the relationship between the function and structure of PFSI was briefly discussed. This is expected to provide some basic insight for the research on the functional effectiveness of PFSI and its applications against HCC in order to establish a theoretical ground for subsequent research on the anti-HCC activity of the purified fractions of PFSI.

## 2. Materials and Methods

### 2.1. Materials

SMMC 7721 HCC cells and human hepatic L02 cells (HL-7702) were purchased from Qingqi Biotechnology Development Co., Ltd. (Shanghai, China); docetaxel (0.5 mL:20 mg; Jiangsu Hengrui Pharmaceutical Co., Ltd.; Jiangsu, China); FSI harvested in August 2021 was authenticated by Zhang Runrong, senior engineer of traditional Chinese medicine, College of Pharmacy and Food Science, Zhuhai College of Science and Technology, Zhuhai, Guangdong Province, China (Production batch: 20210820; Shandong Bozhou R&B Food Sales Co.; Shandong, China); papain (800 U/mg; Shanghai Yuanye Biotechnology Co., Ltd.; Shanghai, China); monosaccharide standards: GulA (Gulonic acid); ManA (Mannuronic acid); Man (Mannose); Rib (Ribose); Rham (Rhamnose); GlcN (Glucosamine); GlcA (Glucuronic acid); GalA (Galacturonic acid); Glc (Glucose); GalN (Galactosamine); Gal (Galactose); Xyl (Xylose); Ara (Arabinose); Fuc (Fucose) (AR; all Sigma-Aldrich(Shanghai) Trading Co., Ltd.; Shanghai, China) 

### 2.2. Methods

#### 2.2.1. Extraction, Separation, and Purification of Polysaccharides

**Figure 1:**
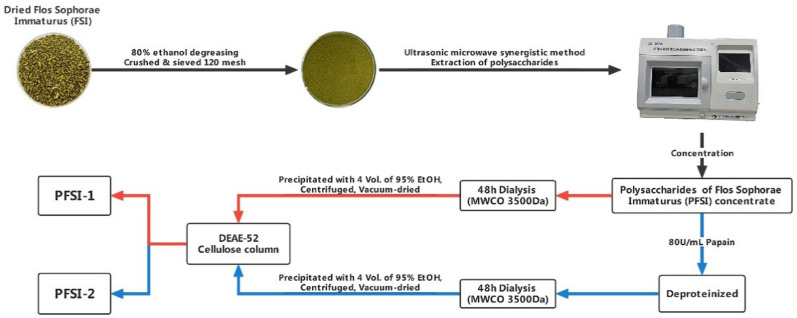
The procedure for the extraction and purification of PFSI-1 and PFSI-2.

An appropriate amount of degreased FSI powder, obtained by oven drying at 60 °C to a constant mass with 120 mesh, was weighed, and distilled water was used as the extraction solution. The PFSI extraction was performed by the ultrasonic–microwave-assisted method, with the parameters of microwave power 500 W, ultrasonic power 270 W, extraction time 21 min, and the ratio of material to liquid 1:95. After cooling at ambient temperature and centrifugation for 10 min (8000 rpm/min), the supernatant was transferred and concentrated. The concentrated solution was placed in an MWCO 3500 Da dialysis bag (due to the selective permeability of the dialysis bag’s semipermeable membrane or the use of its pore size, small molecules such as amino acids and other impurities are filtered out, and the majority of macromolecular polysaccharides are retained, ultimately achieving the purpose of separation) and subjected to running water for 48 h. The concentration was increased by adding 4 times the volume of 95% ethanol before being placed in a refrigerator at 4 °C overnight, followed by washing the lower layer of the precipitate with anhydrous ethanol several times and drying off under reduced pressure. The concentration of 100 mg/mL of the dried product was eluted with 200 mL of distilled water and 0.1 mol/L, 0.2 mol/L, and 0.3 mol/L sodium chloride solutions at a flow rate of 1 mL/min, and then the solutions were pooled and gathered to obtain PFSI-1. The concentration of PFSI concentrate was brought to 80 U/mL, warmed in a constant-temperature water bath at 50 °C for 2 h, removed to a boiling water bath for 10 min for enzyme inactivation, and centrifuged at 6000 rpm/min for 10 min. Then, the precipitate was discarded to obtain the deproteinized solution, and the summary supernatant was collected. The subsequent purification operation was the same as that of PFSI-1 to obtain PFSI-2. The total saccharide content was determined by the phenol sulfuric acid method [24]. The yield of polysaccharides was measured as follows, where Y is the yield of polysaccharide (%), V is the total volume of sample solution (mL), C is the concentration of polysaccharide (mg/mL), D is the dilution factor, and m is the mass of the weighed sample (g). It was calculated that the polysaccharide yield of PFSI-1 after purification was 56.62 ± 1.77% and PFSI-2 was 83.62 ± 1.52%. The procedure for the extraction and purification of PFSI-1 and PFSI-2 is shown in Figure 1.

(1) $$Y\;(\%)=\frac{V\times C\times D}{m\times 10^{3}}\;\times \;100$$

#### 2.2.2. Infrared Spectral Analysis

The fully dried KBr and PFSI-1 and PFSI-2 were blended and ground with an agate mortar in a sufficiently dry environment at a mass ratio of 100:1~200:1. The slices were pressed and scanned with an infrared spectrometer in the range of 400~4000 cm^−1^.

#### 2.2.3. Monosaccharide Composition Determination

We weighed 10–20 mg of PFSI-1 and PFSI-2 polysaccharide samples in a 20 mL jar, added 5 mL of 2 mol/L TFA, sealed the tube with N_2_ (10 L/min, 1 min), and hydrolyzed in an oven at 100 °C for 2 h. The cap was opened after cooling. After hydrolysis, we took 1 mL out and added 1 mL of methanol, then blow-dried with N_2_ under a 70 °C water bath. The adding of methanol and blow-drying with N_2_ was repeated twice to remove TFA; 1 mL of 0.3 mol/L NaOH solution was added to fully dissolve the residue to obtain polysaccharide hydrolysis solution and to ensure dilution for derivatization. Then, 400 μL of mixed monosaccharide standard or polysaccharide hydrolysis solution was added into a 5 mL stoppered test tube, and 400 μL of PMP methanol solution was added, vortexed, and mixed well; the reaction was held in a water bath at 70 °C for 2 h and then cooled to room temperature; 400 μL of 0.3 mol/L HCl was added to neutralize (pH 6~7); 1200 μL of water was applied, and the same volume of chloroform was charged, vortexed, mixed, shaken, and left to stand, and the chloroform phase was discarded. The chloroform phase was withdrawn and evacuated twice. The aqueous phase was filtered through a 0.45 μm microporous membrane (aqueous system) and then fed into the high-performance liquid chromatograph for analysis.

#### 2.2.4. Scanning Electron Microscope (SEM) Analysis

A handful of PFSI-1 and PFSI-2 polysaccharide samples were glued to the bench with conductive adhesive, and, after gold spraying, the surface morphology of the samples was observed under high-vacuum mode, and the morphology of PFSI-1 and PFSI-2 was observed under 100× *g*, 500× *g*, 1000× *g*, 5000× *g*, and 15,000× *g* magnifications.

#### 2.2.5. Atomic Force Microscopy (AFM) Analysis

PFSI-1 and PFSI-2 were measured and prepared in a 0.01 mg-mL-1 solution, which was dropped onto mica flakes, and their molecular patterns were observed by atomic force microscopy after drying.

#### 2.2.6. Nuclear Magnetic Resonance Spectroscopy (NMR) Analysis

We precisely weighed 20 mg samples of dried PFSI-1 and PFSI-2, which were separately dissolved in 0.5 mL of heavy water (D_2_O) with 99.9% purity and then placed in an NMR tube to dissolve fully. The hydrogen (1H) and carbon (13C) spectra of one-dimensional nuclear magnetic resonance (NMR) were detected in the tube utilizing a 500 M nuclear magnetic resonance spectrometer at 25 °C.

#### 2.2.7. Cell Viability Assay

The CCK-8 method [25] was employed to assess cell viability. When SMMC 7721 cells and HL-7702 cells were cultured to the logarithmic growth phase, cell suspensions were derived, which were then inoculated in 96-well plates at a concentration of 2 × 10^5^ cells/mL, with each well adding 100 μL. After incubation in a CO_2_ incubator for 24 h, the cells were plastered and grown all over the bottom of the bottles. The 96-well plate with the old solution aspirated away was divided into an experimental administration group, a positive control group, and a blank control group, and five replicate wells were set up. We adopted the two-fold dilution method to formulate 2000 μg/mL of PFSI-1 and PFSI-2 culture solutions. The 96-well plates were further prepared by filling 100 μL of PFSI and PFSI-2 culture mediums at 125, 250, 500, 1000, and 2000 μg/mL, and 5 replicate wells were set up for each concentration, blank control group, and positive drug group. After incubating in the CO_2_ incubator for 48 h, the medium containing polysaccharides was aspirated and abandoned, followed by adding 100 μL of DMEM medium containing 10% CCK-8 to each well and thermostatically incubating in the CO_2_ incubator for 1.5 h. The absorbance values were measured at 450 nm with an enzyme marker, and the cell proliferation inhibition rate of each group was calculated according to the following equation.

(2) $$\mathrm{Cell}\;\mathrm{proliferation}\;\mathrm{inhibition}\;\%=(1\;-\;OD_{1}/OD_{2})\;\times \;100\%$$

The formula: OD_1_ is the absorbance value of the experimental drug administration group at absorbance 450 nm; OD_2_ is the absorbance value of the blank control group at absorbance 450 nm.

#### 2.2.8. Cell Scratch Wound Healing Assay In Vitro

The method in the literature [26] was based on a 6-well plate, and a marker was used to draw a straight line on the back of the plate. The logarithmically grown SMMC 7721 cells were diluted into a cell suspension of 5 × 10^5^ cells/mL and inoculated into the 6-well plate at 2 mL per well. After 24 h incubation in the CO_2_ incubator, the old solution was aspirated away, and the marked horizontal lines were evenly scored along the bottom of the plate with a 200 μL gun tip perpendicular to the bottom of the plate, and the scored cells were gently washed with PBS. A blank control group, a positive control group, and an experimental administration group were set up, and the positive control group was added with 6.8 μM bevacizumab containing injection solution. The positive control group was added with 6.8 μM bevacizumab injection, and the experimentally administered group was added with 2 mL of PFSI-1 and PFSI-2 culture solutions containing different concentrations and continued to culture. The wells were replicated for 3 groups. The cells were incubated in the CO_2_ incubator for 0 h, 24 h, and 48 h and then observed and photographed under an inverted light microscope, with the area of cell scratches finally calculated using Image J software. The calculation formula is given below. In the formula: S_0_ for 0 h under the scratch space area; S_1_ for 24 h or 48 h under the scratch space area.

(3) $$\mathrm{Cell}\;\mathrm{migration}\;\mathrm{ratio}=(S_{0}\;-\;S_{1})/S_{0}\;\times \;100\%$$

#### 2.2.9. Determination of Apoptosis

The logarithmically grown SMMC 7721 cells were diluted into a cell suspension of 5.0 × 10^5^ cells/mL, and 2 mL per well was inoculated in a 6-well culture plate and incubated in the CO_2_ incubator until the cells grew to more than 80% density of the occupied well area; then, the culture medium was pipetted and disposed of, and 2 mL of PFSI-1 and PFSI-2 medium containing different concentrations were continuously incubated. After culturing for 48 h in the CO_2_ incubator, the PFSI-1 and PFSI-2 cultures containing 250 μg/mL, 500 μg/mL, and 1000 μg/mL were aspirated, and the original culture medium was collected, then digested by adding 500 μL of 0.25% trypsin for 2 min, and the digestion was terminated by adding 500 μL of the serum-containing medium, and the cell suspension was gently blown and transferred to a 1.5 mL centrifuge tube. We centrifuged the cell suspension at 1500 rpm for 5 min, gently aspirated the supernatant with a pipette, washed twice with PBS solution, then added 500 μL of 1× binding buffer to resuspend the cells, centrifuged at 1500 rpm for 5 min at 4 °C, then centrifuged and discarded the supernatant. We resuspended the cells with 100 μL × binding buffer, added 5 μL Annexin V-FITC, mixed gently and kept away from light at room temperature for 10 min, added 5 μL PI, kept away from light at room temperature for 2 min, added 400 μL PBS solution, and immediately detected by flow cytometry.

#### 2.2.10. Investigation of Cell Cycle

By referring to the instructions and literature [27], logarithmically grown SMMC 7721 cells were diluted into a cell suspension of 5.0 × 10^5^ cells/mL and inoculated in 6-well culture plates at 2 mL per well, incubated in the CO_2_ incubator until the cells grew to a density of 80% or more of the occupied well area, and then the culture medium was aspirated and discarded. For the negative control group and experimental drug administration group, 2 mL of PFSI-1 and PFSI-2 culture medium without drugs and at concentrations of 250 μg/mL, 500 μg/mL, and 1000 μg/mL were used for continued incubation. After 48 h incubation in the CO_2_ incubator, the old culture medium was aspirated and collected, 400 μL of low-concentration trypsin was added to digest for 1 min, 400 μL of the serum-containing medium was included to terminate the digestion, and the cell suspension was gently blown and transferred to a 1.5 mL centrifuge tube and centrifuged at 1500 rpm for 5 min. The supernatant was gently aspirated with a pipette gun and washed once with PBS solution, the cell concentration was adjusted to 1 × 10^6^ cells/mL, and 1 mL of single-cell suspension was taken. After centrifugation, we removed the supernatant, added 500 uL of 70% precooled ethanol to the cells for 2 h overnight, stored at 4 °C, and washed off the fixation with PBS before staining. We added 100 µL of RNase A solution to the cell precipitate, resuspended the cells, and immersed them in a water bath at 37 °C for 30 min. Then, we inserted 400 µL of PI staining solution and incubated for 30 min at 4 °C, avoiding light. The red fluorescence at the excitation wavelength of 488 nm was recorded.

#### 2.2.11. Statistical Analysis

Data are shown as mean ± SEM, and statistical analysis was performed using Origin 2021, Graphpad Prism 8, Image J, and EXCEL 2019. To compare the differences between multiple groups, a one-way analysis of variance (ANOVA) was performed in this software. *p* < 0.05 indicates statistical significance.

## 3. Results

### 3.1. Monosaccharide Composition of Analysis

**Figure 2:**
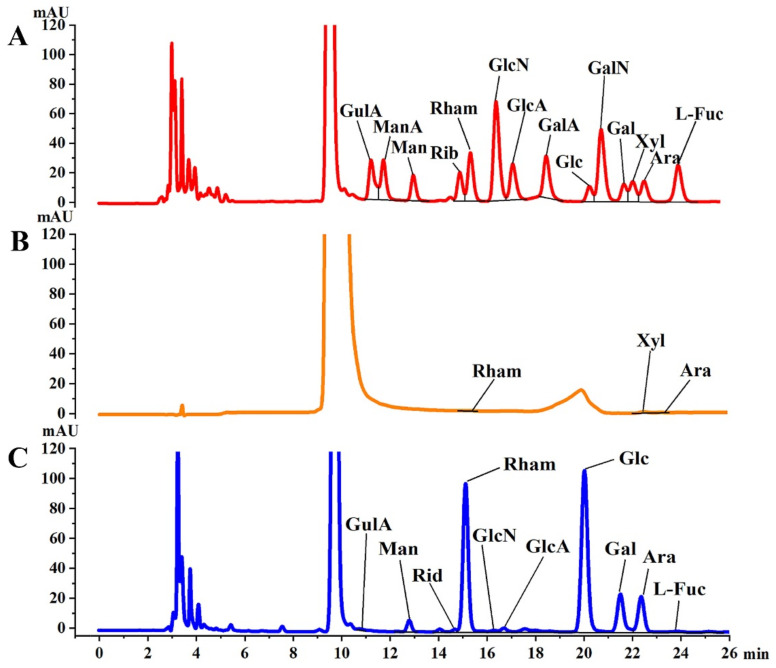
Total ionic flow diagram of standard monosaccharide chromatogram (**A**), PFSI-1 (**B**), and PFSI-2 (**C**) of monosaccharide.

Figure 2A shows the liquid chromatographic analysis of the monosaccharide standards, and the peaks in order of retention time were GulA, ManA, Man, Rib, Rham, GlcN, GlcA, GalA, Glc, GalN, Gal, Xyl, Ara, Fuc. PFSI-1 and PFSI-2 were hydrolyzed with complete acid to prepare the glycolonitrile derivatives. Chromatograms after liquid chromatographic analysis are shown in Figure 2B, and C. According to Table 1, PFSI-1 contained three monosaccharides, namely Rham, Xyl, and Ara, with molar ratios of 8.28%, 70.10%, and 21.62%, respectively, and PFSI-2 contained ten monosaccharides, namely GulA, Man, Rib, Rham, GlcN, GlcA, Glc, Gal, Ara, and Fuc, with molar ratios of 0.04%, 2.19%, 0.44%, 16.95%, 0.07%, 0.48%, 58.01%, 11.62%, 10.00%, and 0.20%, respectively. Given the fact that monosaccharides such as rhamnose and arabinose have certain biological activities [28], such as hypolipidemic, hypoglycemic, and other biological functions, it is presumed that PFSI-1 and PFSI-2 have certain biological activities.

### 3.2. IR of Analysis

**Figure 3:**
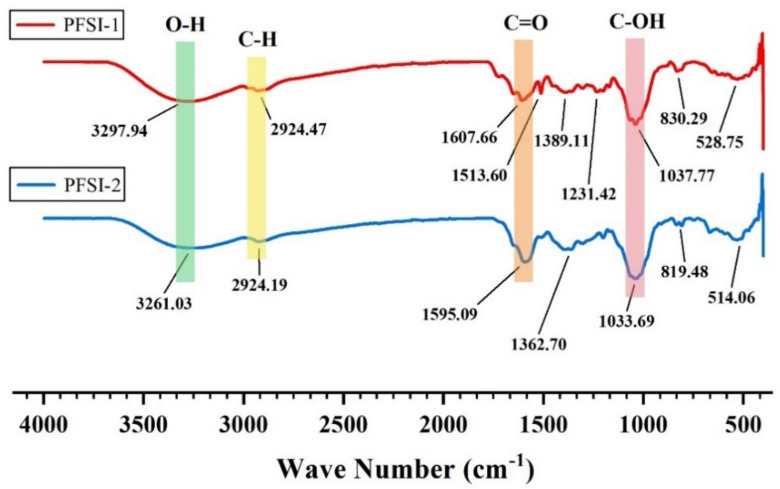
Infrared spectra of PFSI-1 and PFSI-2.

The infrared spectra of PFSI-1 and PFSI-2 are shown in Figure 3. It can be seen that there are absorption peaks in 3600~3000 cm^−1^, 2900 cm^−1^, 1600~1400 cm^−1^, and 1200~700 cm^−1^, both on the left and right side, and the absorption peaks in these regions are typical for polysaccharides. The absorption peaks at 3261 cm^−1^ and 3297 m^−1^ indicate that the polysaccharide may exist. The absorption peaks of PFSI-1 and PFSI-2 with wider and stronger peaks are O-H functional group absorption peaks, which are generated by the hydroxyl groups on the sugar chains of PFSI-1 and PFSI-2. As there are a large number of hydroxyl groups on the polysaccharide chains, which can form intramolecular as well as intermolecular hydrogen bonding, the waveforms of the peaks are broad, and the wave numbers move to lower frequencies. The weaker absorption peak around 2924 cm^−1^ is caused by the C-H stretching vibration. The sharper absorption peaks around 1595 cm^−1^ and 1607 cm^−1^ are the C=O stretching vibrations and N-H variable angle vibrations, indicating absorption peaks caused by asymmetric carbonyl stretching. In combination with the UV spectrum, the absorption peaks appeared due to the stretching vibration of the carboxylate ion (-COO-) in the sample. The absorption peaks at 1389 cm^−1^ and 1362 cm^−1^ are due to the stretching vibration of C=C. The relatively large absorption peaks at 1037 cm^−1^ and 1033 cm^−1^ are due to the C-OH stretching vibration [29]. In addition, the characteristic absorption peaks at around 819 cm^−1^, and 830 cm^−1^ are not only characteristic of α-type differential isomers but are also caused by the stretching vibration of C=O on the pyranose ring, indicating that the sugar chain of the sample contains a pyranose ring structure, confirming the presence of a-D-pyranose (C_1_-H) as well as a- and β-configuration glycosidic bonds. From the analysis of each absorption peak before 1000 cm^−1^, it can be initially inferred that the tested sample is a sugar structure. The results of infrared spectra showed that PFSI-1 and PFSI-2 have functional groups of polysaccharides and are typical polysaccharides.

### 3.3. SEM of Analysis

**Figure 4:**
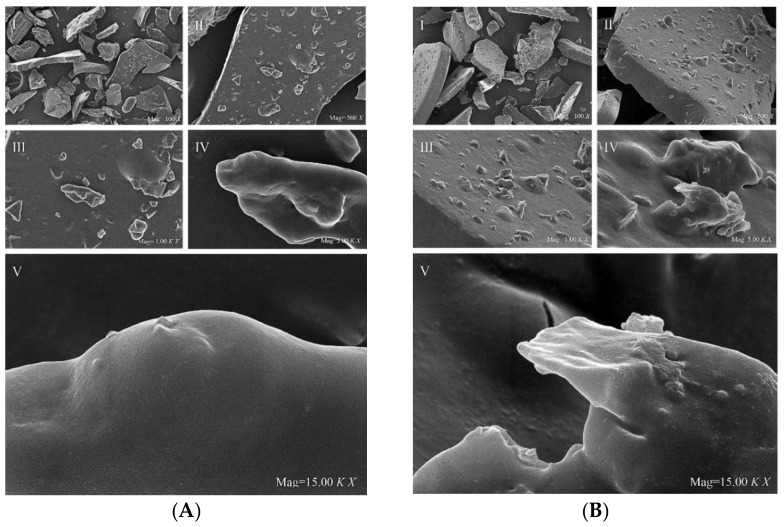
SEM images of PFSI-1 (**A**), PFSI-2 (**B**). (Note: Polysaccharide features viewed in PFSI-1 (**A**) and PFSI-2 (**B**) I at 100× magnification; II, III at 500× and 1000× magnification microscopy; IV, V at 5000× and 15,000× microscopy.)

Figure 4I shows PFSI-1 (A) and PFSI-2 (B) at 100×, from which clear and structurally complete polysaccharides with a miscellaneous dense constituent composition and a blocky structure can be observed. At 500× and 1000× microscopy in Figure 4II,III, it can be seen that it presents a clear fragmented structure with an irregular lamellar solid morphology and a tight structure, and no obvious voids inside the polysaccharide, which may be related to the larger molecular weight of the polysaccharide. In this case, the surface of PFSI-1 and PFSI-2 was smooth and bulged like spheres, indicating that they might have a complex structure, similar to the ginkgo seed polysaccharide in the literature [30], which has some stability, as revealed by Figure 4IV,V under 5000× and 15,000× magnifications.

### 3.4. AFM of Analysis

**Figure 5:**
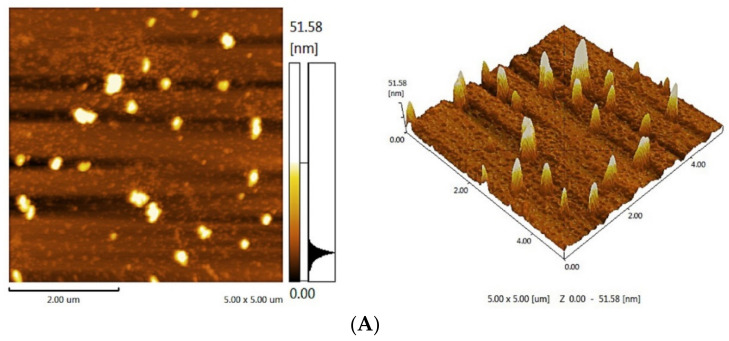

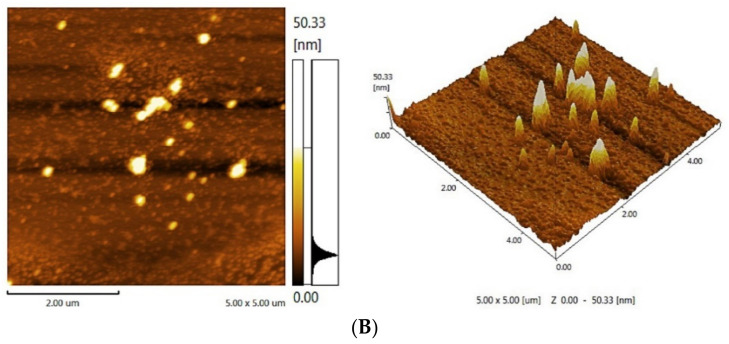
AFM diagram of PFSI-1 (**A**) and PFSI-2 (**B**).

The AFM planar and cubic spectra of PFSI-1 and PFSI-2 are illustrated in Figure 5 below. In the planar spectrograms, multiple molecular chains are entangled together to form irregular sheets, and the heights of these irregular sheet-like monomers are in the range of 0~51.58 nm. On the other hand, one can see that both PFSI-1 and PFSI-2 show irregular ellipsoidal and cylindrical-like structures, which are similar to those observed by SEM. In general, the single-chain diameters of polysaccharides are in the range of 0.1~1 nm; as can be seen from the figure, the diameters of PFSI-1 and PFSI-2 are well above this range, suggesting that what is observed by AFM is not a single sugar chain molecule. The cubic spectrogram suggests that the glycan chains are entangled due to the presence of a large number of hydroxyl groups in the polysaccharide or due to the presence of van der Waals forces between the polysaccharide molecules, resulting from the irregular aggregation of polysaccharide chain molecules.

### 3.5. NMR of Analysis

**Figure 6:**
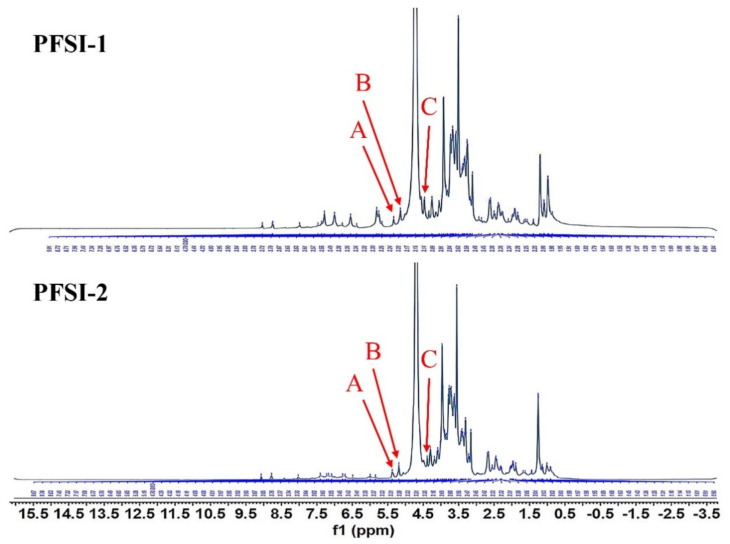
PFSI-1, PFSI-2 NMR hydrogen spectra (1H NMR). Note: PFSI-1 and PFSI-2 both had 3 heterotopic hydrogen signals at δ5.31 (A), 5.12 (B), and 4.52 (C) ppm and δ5.38 (A), 5.19 (B), and 4.49 (C) ppm in 1H-NMR, respectively, and all consisted of three kinds of saccharide residues with glycosidic bonds comprising β and α types.

According to Figure 6 PFSI-1 and PFSI-2 both had three heterotrophic hydrogen signals in 1H-NMR at δ 5.31, 5.12, and 4.52 ppm as well as δ 5.38, 5.19, and 4.49 ppm, implying that the chemical shift signals that appeared at these three sites were PFSI and PFSI-2 and were composed of three types of saccharide residues, and the glycosidic bonds contained β and α types. Given the analysis of the composition of the monosaccharides and the relevant literature [28], it could be β-D-fructose and α-L-rhamnose. The constitution of the saccharide ring can also be inferred from the coupling constant (J) of the heterotrophic proton to the neighbor proton; when J is 9.6 Hz, the heterotrophic proton is in the perpendicular bond directly to the adjacent proton. The chemical shift of the H-1 proton was found to be in excess of 4.95 ppm, which allowed us to determine the α-pyranose conformation of the saccharide ring, which is consistent with the findings of the IR pattern.

**Figure 7:**
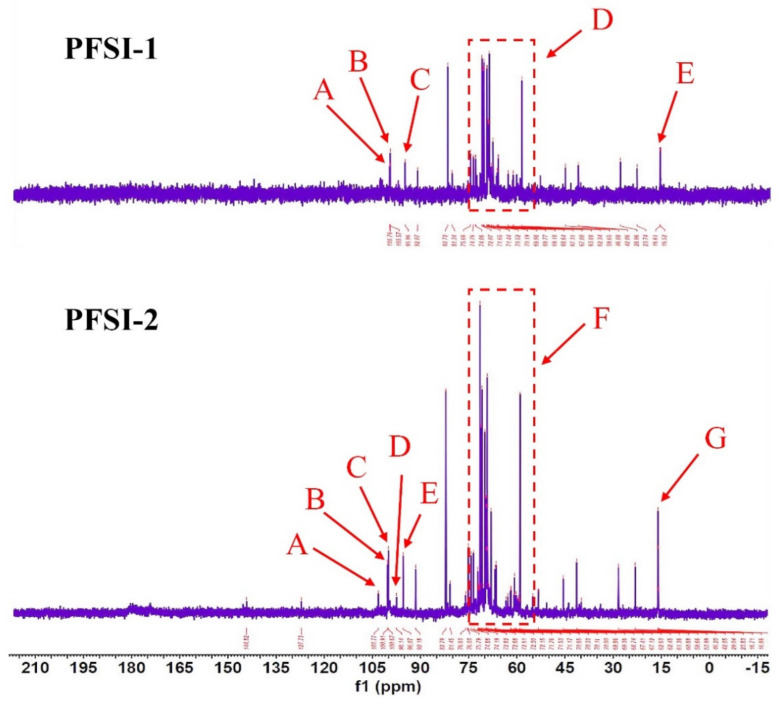
PFSI-1, PFSI-2 NMR carbon spectra (13C NMR). Note: PFSI-1 and PFSI-2 are mainly present with α-configuration saccharide groups. The chemical shift signals appeared as heterocapsid carbon signal peaks with δ 100.76 (A), 100.57 (B), 95.96 (C) ppm and δ 103.77 (A), 100.91 (B), 100.67 (C), 98.14 (D), 96.07 ppm, respectively. The δ values of C_2_ to C_5_ signals in PFSI-1 (part D) and PFSI-2 (part F) are all concentrated between δ 65 and 77, indicating that most of them have not been replaced yet. There are chemical shifts between δ 55 and 65, illustrating that the hydroxyl group at position C6 is partly substituted and partly unsubstituted. The chemical shifts at δ 16.52 (E) and δ 16.55 (G) in PFSI-1 and PFSI-2 are the C_6_ of rhamnose.

This is illustrated in Figure 7, as 13 C-NMR has a broader range of chemical shifts than 1 H-NMR, which is up to 300 ppm and has a superior definition, in addition to the carbon spectral data for monosaccharides, oligosaccharides, and polysaccharides which are available in much of the literature and can be exploited for comparison to determine the chemical shifts of individual carbons. In the same way, as in 1 H-NMR, the heterotrophic carbon of sugars was located in the lower ranges, PFSI-1, and PFSI-2 at δ 95~110 ppm, with three and five signals in this span, and the chemical shift signals occurring as heterotrophic carbon signal peaks at δ 100.76, 100.57, and 95.96 ppm as well as δ 103.77, 100.91, 100.67, 98.14, and 96.07 ppm. It indicates that the α-configuration glycosyl group has mainly existed in PFSI-1 and PFSI-2. In addition, the δ values of C_2_ to C_5_ signals are all concentrated between δ 67 and 77, which means that most of them were not yet substituted. There were chemical shifts between δ 55 and 65, accounting for the substitution of partially substituted and unsubstituted hydroxyl groups at the C6 position. We additionally detected that the chemical shifts at δ 16.52 and δ 16.55 in PFSI-1 and PFSI-2 are assigned to the C_6_ of rhamnose [31]. This was in agreement with the analysis of the IR and major monosaccharide composition outcomes.

### 3.6. Cell Viability Assay Analysis

#### 3.6.1. Proliferation Inhibition of SMMC 7721 Cells

**Figure 8:**
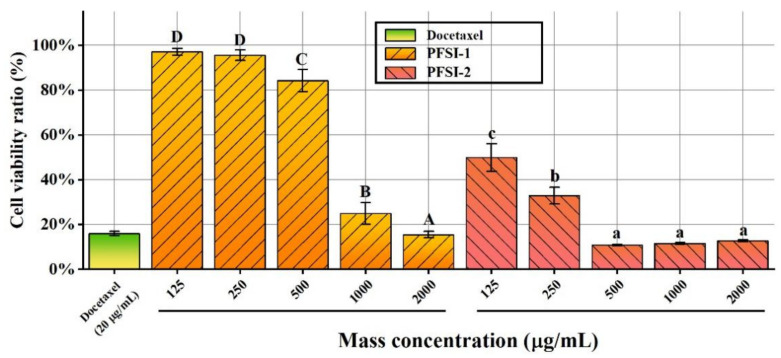
Effect of PFSI-1 and PFSI-2 on the viability of SMMC 7721 cells. (Note: the same small (capital) letters indicate no significant difference between groups, and different small (capital) letters indicate a significant difference between groups, *p* < 0.05).

The influence of PFSI-1 and PFSI-2 on the vitality of SMMC 7721 cells is depicted in Figure 8. With the increase in PFSI-1 and PFSI-2 concentrations, there was a significant inhibition of the proliferation of SMMC 7721 cells (*p* < 0.05). When the PFSI-1 concentration was 125–250 μg/mL, there was no significant difference in the inhibitory effect on the proliferation of SMMC 7721 cells, and the density at this point had not reached the proliferation-inhibiting effective concentration of the cells. When the PFSI-1 concentration was 500–2000 μg/mL, there was a significant quantitative effect on the proliferation inhibition of SMMC 7721 cells (*p* < 0.05); when the PFSI-1 concentration was 2000 μg/mL, the cell survival rate reached a minimum of 15.44%. When the PFSI-2 concentration was 125–500 μg/mL, the inhibition of proliferation of SMMC 7721 cells was concentration-dependent (*p* < 0.05); when concentration was 500–2000 μg/mL, there was no significant difference in the inhibition of proliferation of the cells, indicating that the maximum effect had been reached, and the inhibition was similar to that of the docetaxel control. Compared with the docetaxel control, the suppressive role was similar and the viability of SMMC 7721 cells attained a minimum of 10.77%.

#### 3.6.2. Proliferation Inhibition of HL-7702 Cells

**Figure 9:**
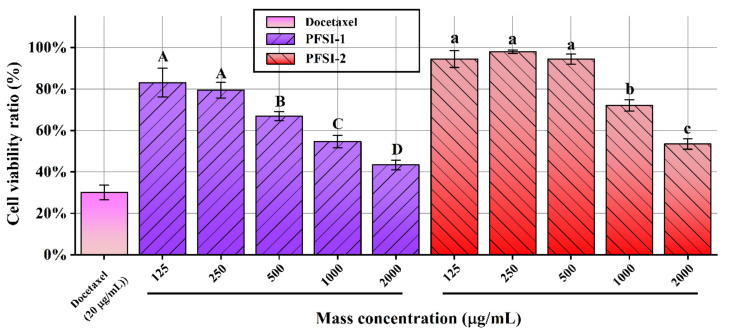
Effects of PFSI-1 and PFSI-2 on the viability of HL-7702 cells. (Note: the same small (capital) letters indicate no significant difference between groups, and different small (capital) letters indicate a significant difference between groups, *p* < 0.05).

The impacts of PFSI-1 and PFSI-2 on the viability of HL-7702 cells are shown in Figure 9. We can see that, with the increase in polysaccharide concentration, PFSI-1 and PFSI-2 have a marked function in inhibiting the proliferation of HL-7702 cells. When the concentration of PFSI-1 was 125–250 μg/mL, there exists no significant difference in the inhibitory function of PFSI-1 on the proliferation of HL-7702 cells, and the concentration of PFSI-1 at this time did not reach the effective concentration to restrain the proliferation of the cells. When the PFSI-1 concentration was 500–2000 μg/mL, the inhibitory effect on the proliferation of HL-7702 cells was quantitatively significant; when it’s concentration was 2000 μg/mL, the survival rate of the cells reached a minimum of 43.39%, which was statistically significant (*p* < 0.05). When the PFSI-2 concentration was 125–500 μg/mL, there was no significant difference in the inhibitory effect on the proliferation of HL-7702 cells, and the PFSI-2 concentration at this juncture failed to achieve the effective concentration for inhibiting the proliferation of the cells, showing that PFSI-2 at 125–500 μg/mL concentration had no inhibitory effect on the cells and that a potential hepatoprotective effect may exist at low concentrations. In contrast, PFSI-2 showed a greater protective effect on HL-7702 cells than PFSI-1 at 125–500 μg/mL; when PFSI-2 concentration was 1000–2000 μg/mL, proliferation inhibition of the cells was quantitatively effective, and the cell survival rate reached a minimum of 53.44%; although, for HL-7702 cells at somewhat high concentrations, there was some inhibition of proliferation. When compared with the docetaxel positive control, the protective efficacy of HL-7702 cells at low concentrations was found to be statistically significant (*p* < 0.05).

### 3.7. Cell Scratch Wound Healing Assay In Vitro Analysis

**Figure 10:**
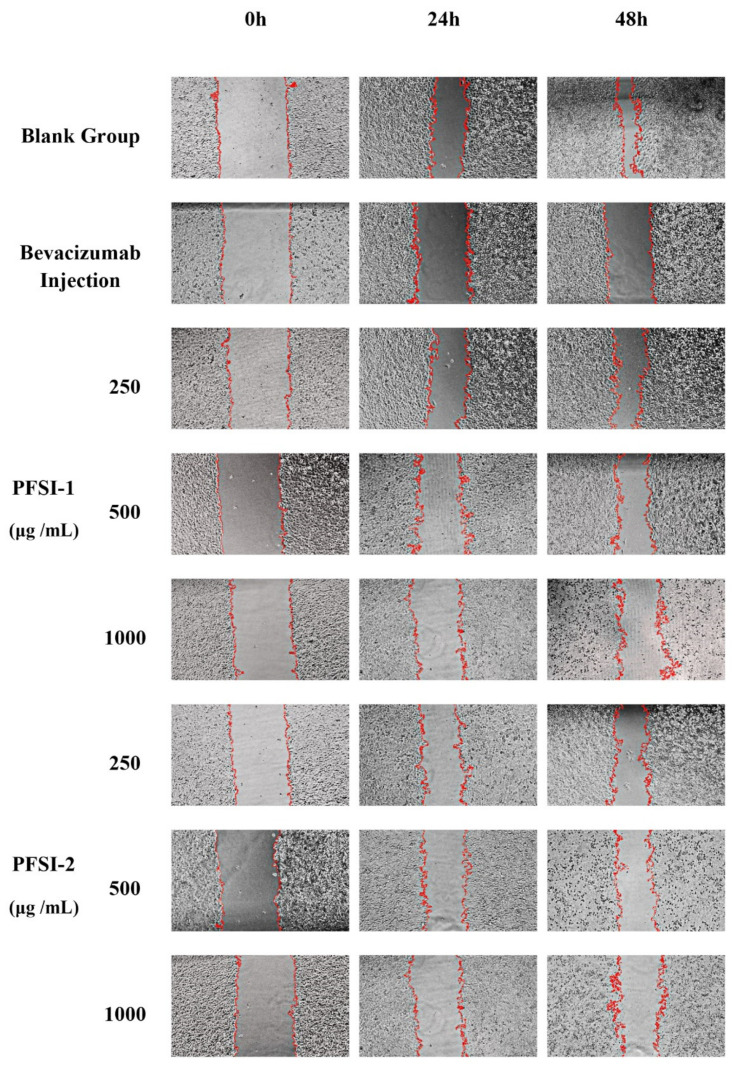
Scratching results of PFSI-1 and PFSI-2 on SMMC 7721 cells.

**Figure 11:**
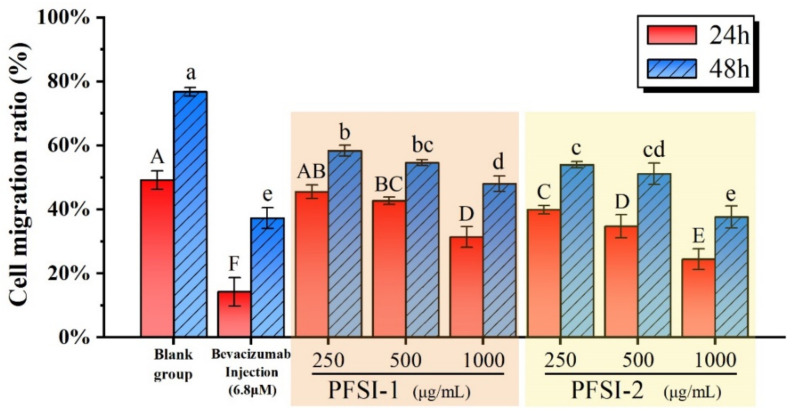
Migration ability of PFSI-1 and PFSI-2 on SMMC 7721 cells. (Note: the same small (capital) letters indicate no significant difference between groups, and different small (capital) letters indicate a significant difference between groups, *p* < 0.05).

The influences of PFSI-1 and PFSI-2 on the migratory ability of SMMC 7721 cells are displayed in Figure 10 and Figure 11. The experimental administration group and positive control group significantly inhibited the migration of SMMC 7721 cells compared with the blank control group (*p* < 0.05). With the increase in PFSI-1 and PFSI-2 concentrations, the migration of SMMC 7721 cells was lower, and all had a certain ability to inhibit the migration of the cells. When the incubation time was 24 h, the minimum migration rate was reached at the concentration of 1000 μg/mL for PFSI-1 and PFSI-2, which were 31.33 ± 3.22% and 24.40 ± 3.22%, depending on the concentration ofthem, respectively. When the incubation time was 48 h, the mobility of PFSI-1 and PFSI-2 both reached the minimum at a concentration of 1000 μg/mL with 47.99 ± 2.44% and 37.57 ± 3.44% for PFSI-1 and PFSI-2, respectively. and were statistically significant (*p* < 0.05).

### 3.8. Determination of Apoptosis Analysis

**Figure 12:**
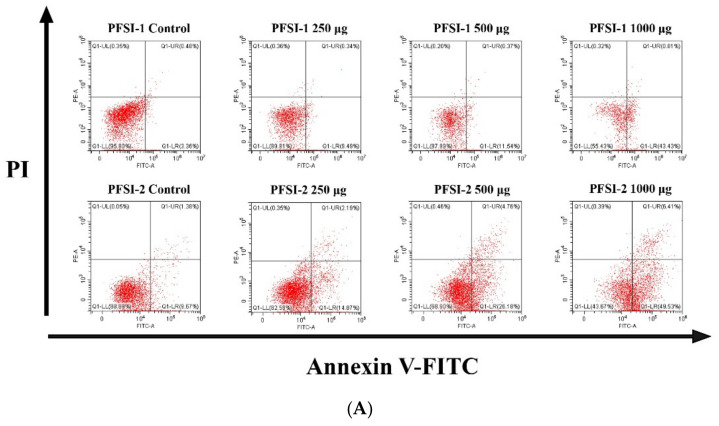

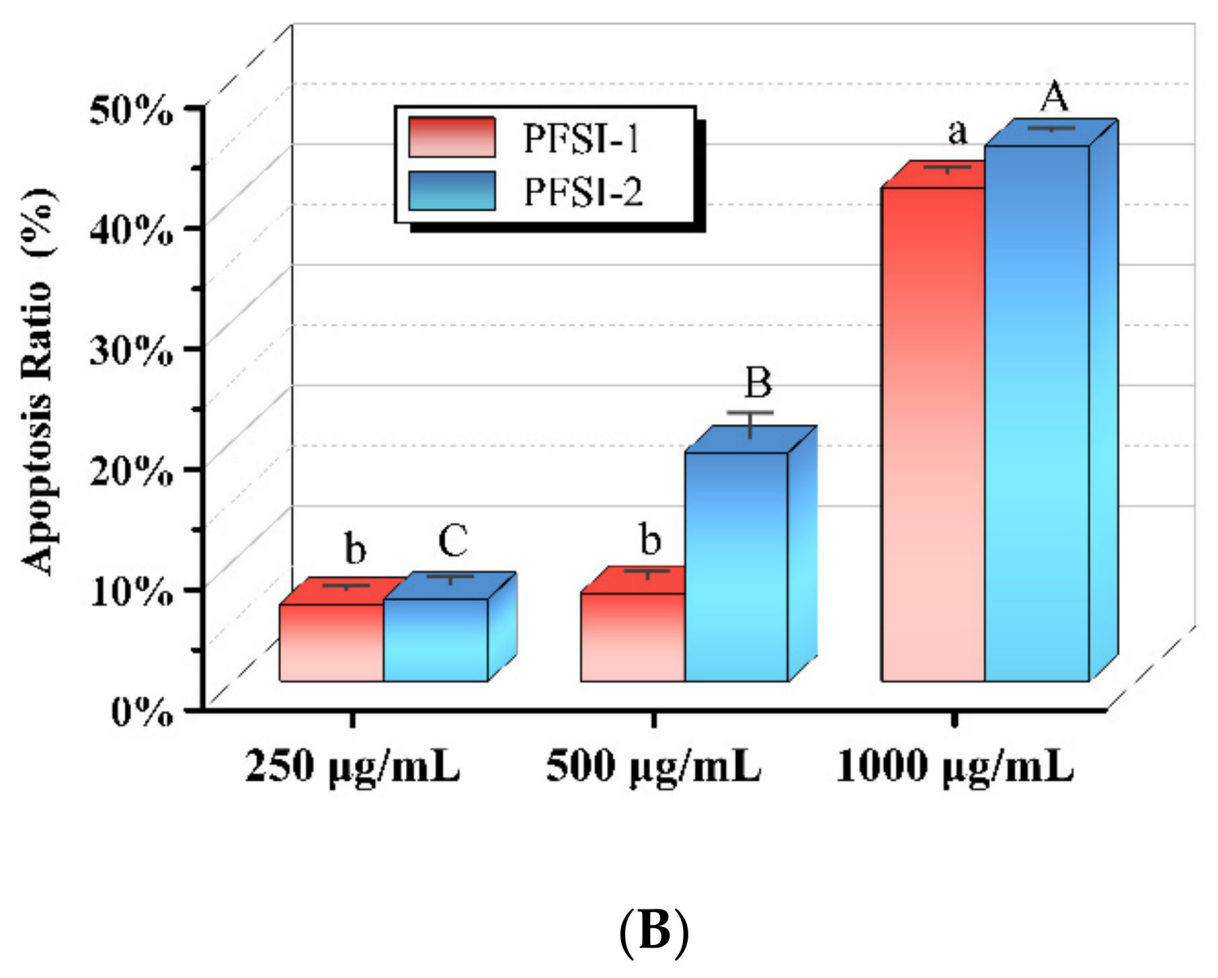
Effect of PFSI-1 and PFSI-2 on apoptosis ratio of SMMC 7721 cells. (Note: (**A**) represents the summary graph of PFSI-1 and PFSI-2 at apoptotic levels in SMMC 7721 cells as detected by flow cytometry; (**B**) represents histograms of the levels of apoptosis in SMMC 7721 cells at different concentrations of PFSI-1 and PFSI-2; the same small (capital) letters indicate no significant difference between groups, and different small (capital) letters indicate a significant difference between groups, *p* < 0.05).

The relationships between PFSI-1 and PFSI-2 on the apoptosis level of SMMC 7721 cells are shown in Figure 12A,B. The incorporation of different concentrations of PFSI-1 and PFSI-2 significantly promoted the apoptosis of SMMC 7721 cells compared with the blank control group (*p* < 0.01). A higher apoptosis ratio of SMMC 7721 cells was noted with the increase in PFSI and PFSI-2 concentrations. At the concentration of 1000 μg/mL, the apoptosis ratio of PFSI-1 and PFSI-2 on SMMC 7721 cells was 44.24% and 55.94%, as well as statistically significant (*p* < 0.05).

### 3.9. Determination of Cell Cycle

**Figure 13:**
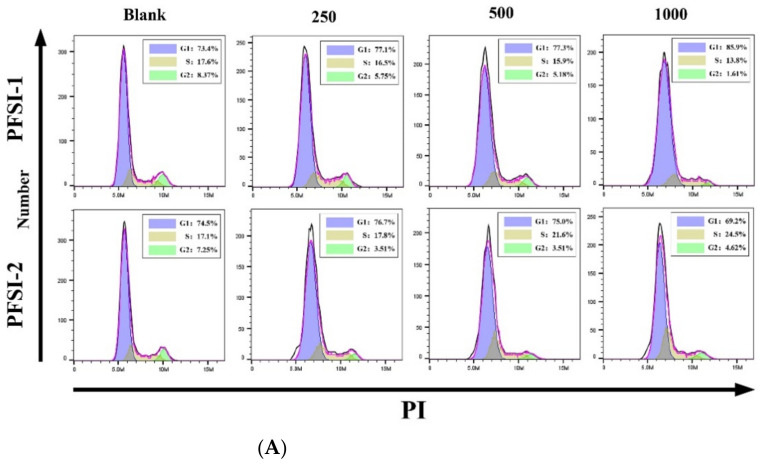

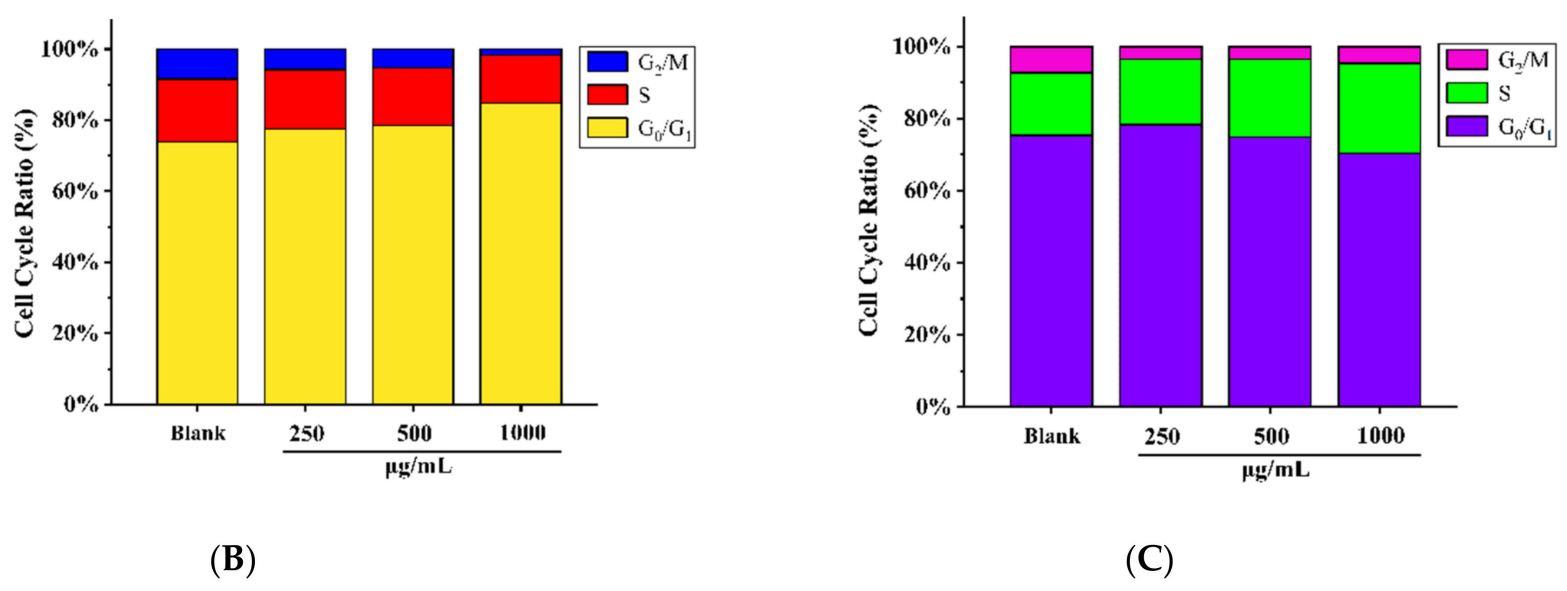
The influence of PFSI-1 and PFSI-2 on the cell cycle of SMMC 7721. (Note: (**A**) represents the red fluorescence at 488 nm recorded on the flow cytometer of PFSI-1 and PFSI-2 on SMMC 7721 cells; (**B**) represents PFSI-1 on SMMC 7721 cell cycle levels; (**C**) represents PFSI-2 on SMMC 7721 cell cycle levels).

The impact of PFSI-1 and PFSI-2 on SMMC 7721 cell cycle levels is demonstrated in Figure 13A–C. Compared with the blank control group, the differences were found to be statistically significant (*p* < 0.05) between the G0/G1-phase and S-phase cell groups. Among them, G0/G1-phase cells increased and S-phase cells decreased in the PFSI-1 group; G0/G1-phase cells decreased, S-phase cells increased, and the apoptosis rate decreased in the PFSI-2 group (*p* < 0.05). The comparisons of G2/M-phase cells in each group showed no statistically significant differences (*p* > 0.05).

## 4. Discussion

**Figure 14:**
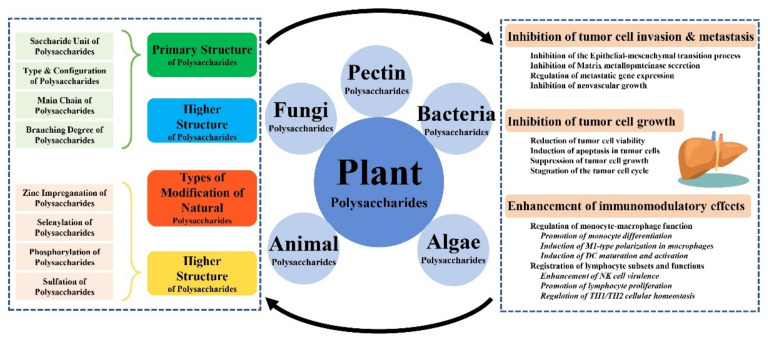
Antitumor mechanism of plant polysaccharides and their conformational interactions.

Plant polysaccharides have become a research hotspot for food and pharmaceuticals owing to their excellent biocompatibility. The anticancer efficacy of polysaccharides derived from Chinese medicine constitutes a complex process, and the multiple anticancer mechanisms of polysaccharides are independent and interrelated [32]. The antitumor effects of plant polysaccharides and their associated linkages are shown in Figure 14. Several surveys have shown that polysaccharides perform cancer-fighting functions through a variety of mechanisms, which are both independent and interrelated. Currently, these studies focus on the tumor-suppressive properties of natural polysaccharides, including the regulation of the cell cycle, apoptosis-related genes, and signaling pathways; effects on tumor angiogenesis and metastasis; and the enhancement of immune function [33]. In particular, the variability of polysaccharide structures, such as the position of monosaccharide residues, the placement of glycosidic bonds, and the sequence of monosaccharide residues, is intimately related to biological activity. Modification of the original structure of plant polysaccharides can potentiate the antitumor activity of polysaccharides. It is crucial to further dissect the functional–structural relationships of plant polysaccharides based on their molecular weight, glycosidic bond typology, monosaccharide composition, alternative modifications, and advanced structures to investigate the conformational relationships of antitumor plant polysaccharides [34,35].

Numerous studies have found that the antitumor activeness of plant polysaccharides is closely related to their molecular weight, monosaccharide composition, advanced structure, and other structural relationships. The mechanism of the action of plant polysaccharides does not differ significantly among different families and is multifaceted: it can achieve antitumor effects by modulating the metabolism of iron and energy, enhancing immune regulation, affecting the expression of genes and related proteins, and impeding tumor angiogenesis, invasion, and metastasis. Its antitumor activity is closely related to the conformation of the primary and advanced structure, substituents, and molecular mass of the polysaccharide [36,37]. The signaling pathways involved in the development of tumors are intricate and interrelated, and so further research into the signaling pathways through which plant polysaccharides exert their antitumor effects would be a tremendous boost to their development and application. However, less research has been accomplished on the antitumor and advanced structure–activity relationships of plant polysaccharides in vivo. In contrast, the antitumor activation of plant polysaccharides relates well to their molecular weight, monosaccharide composition, and glycosidic linkage. Further exploration of the complete molecular weight range and monosaccharide species of plant polysaccharides that exert antitumor activity is of great relevance to unraveling their structure–activity associations and facilitating their development.

At present, with the increasingly high-speed pace of modern life, the number of people suffering from HCC remains at high levels. Thus, there is a necessity to discern and develop high-efficiency and low-toxicity drugs for the treatment of HCC. Since synthetic drugs have their inevitable disadvantages, natural products derived from traditional natural herbal medicines have caught the attention of researchers in recent years. In this study, PFSI was extracted by ultrasonic microwave extraction and purified without deproteinization to obtain PFSI-1 and purified by papain preparation to obtain PFSI-2. The antitumor activities of PFSI-1 and PFSI-2 were analyzed for comparison, and it was found that them could significantly inhibit the proliferation of SMMC 7721 cells, and PFSI-2 had a better suppressive function. In addition, by investigating the inhibitory effects of PFSI-1 and PFSI-2 on the proliferation of human hepatic L02 cells (HL-7702), it was found that PFSI-2 inhibited the cells less than PFSI-1, revealing that PFSI-2 at less than 500 μg/mL has some potential to protect the liver. This may be related to the difference in the degree of polysaccharide purification, molecular weight, molecular structure, conformation, monosaccharide composition, and so on.

The structural characterization of PFSI-1 and PFSI-2 showed that they had characteristic absorption peaks of polysaccharides and represented a typical polysaccharide sample. The results showed that PFSI-1 contained three monosaccharides, namely Rham, Xyl, and Ara, with molar proportions of 8.28%, 70.10%, and 21.62%, respectively, and that PFSI-2 contained ten monosaccharides, namely GulA, Man, Rib, Rham, GlcN, GlcA, Glc, Gal, Ara, and Fuc. The molar ratios of PFSI-2 were 0.04%, 2.19%, 0.44%, 16.95%, 0.07%, 0.48%, 58.01%, 11.62%, 10.00%, and 0.20%, respectively. Given the fact that rhamnose and arabinose have certain biological activities such as hypolipidemic, hypoglycemic, and other biological functions that have been reported in existing studies, it is presumed that PFSI-1 and PFSI-2 have many biological activities that are worth exploring. There are monosaccharides shared by PFSI-1 and PFSI-2, and it can be speculated that they may be an important component for exerting activity. In addition, SEM and AFM observations showed that PFSI-1 and PFSI-2 had irregular ellipsoidal and cylindrical-like structures, suggesting that PFSI may have a complex structure, indicating that it may have more active potential. In future studies, the isolation and purification of PFSI will be continued, and the classification of tumor activity will be explored from the monosaccharide composition, etc., to augment new therapeutic avenues for glycans in the field of cancer management.

## 5. Conclusions

A first extraction, isolation, and structural characterization of PFSI were carried out in the proposed study. In summary, the cytotoxic effects of PFSI-1 without deproteinization and PFSI-2 with papain treatment on SMMC 7721 cells were also investigated. It was found that PFSI-1 and PFSI-2 had a clear inhibitory effect on SMMC 7721 cells in a concentration-dependent mode and that the weaker the migration ability of SMMC 7721 cells, the higher the rate of promoting apoptosis and blocking G0/G1-phase and S-phase at the cell-cycle level. Moreover, PFSI-2 has some hepatoprotective potential against human hepatic L02 cells (HL-7702) at less than 500 μg/mL. Consequently, PFSI promises to be a potential new drug with low toxicity and effective activators to directly or indirectly adjunctively to treat HCC.

## Figures and Tables

**Table 1:** Molar percentages of monosaccharide components of PFSI-1 and PFSI-2. (Note: - represents no detections).

Monosaccharides	Molar Percentage %	
	PFSI-1	PFSI-2
Gulonic acid	-	0.04
Mannuronic acid	-	-
Mannose	-	2.19
Ribose	-	0.44
Rhamnose	8.28	16.95
Glucosamine	-	0.07
Glucuronic acid	-	0.48
Galacturonic acid	-	-
Glucose	-	58.01
Galactosamine	-	-
Galactose	-	11.62
Xylose	70.10	-
Arabinose	21.62	10.00
Fucose	-	0.20

## Data Availability

Not applicable.

## Sample Availability

Samples of the compounds are not available from the authors.
